# Evaluating advance peace in Fresno, California: An interrupted times series analysis of a community-based gun violence intervention

**DOI:** 10.1371/journal.pone.0328780

**Published:** 2025-08-27

**Authors:** Xing Gao, Juan R. Cabrera, David Padilla, Mahasin S. Mujahid, Jason Corburn

**Affiliations:** 1 Department of Obstetrics, Gynecology, & Reproductive Sciences, University of California San Francisco, San Francisco, California, United States of America; 2 Division of Epidemiology, School of Public Health, University of California Berkeley, Berkeley, California, United States of America; 3 Department of City and Regional Planning and the School of Public Health, University of California Berkeley, Berkeley, California, United States of America; Yale University, UNITED STATES OF AMERICA

## Abstract

**Background:**

Gun violence is a critical public health issue, contributing to the disproportionate burden of health inequities among racially and economically marginalized populations. Advance Peace, a community-driven gun reduction program that integrates street outreach workers to interrupt conflicts with trauma-informed programming to provide mentorship and support for young people at the center of urban gun violence, may be a strategy to reduce gun violence and build healthy communities. We assessed whether the implementation of Advance Peace in Fresno, California was associated with a reduction in gun-related violence, including homicides and assaults. We hypothesized that post-implementation of Advance Peace, there would be a reduction in both gun-related homicides and assaults.

**Methods:**

Leveraging crime statistics from the Fresno Police Department on gun-related homicides and assaults between January 2014 and June 2023, we evaluated the impact of Advance Peace programming, implemented beginning in July 2021, on gun violence in Fresno. Descriptive analysis assessed average gun violence rates over time. We used interrupted time series models to assess the rates of gun violence associated with the implementation of Advance Peace in Fresno.

**Results:**

In Fresno, there was evidence of a reduction in crime rates following the introduction of Advance Peace intervention. Two years post-intervention, there was a 46% decrease in the rate of all gun-related crimes, including both homicides and assaults (rate ratio: 0.54, 95% CI: 0.36–0.81). The intervention was also associated with a reduction in the rate of gun-related homicides (RR = 0.45, 95% CI: 0.21–0.95) and the rate of gun-related assaults (RR = 0.58, 95% CI: 0.38–0.89).

**Conclusion:**

Findings from this study demonstrate that Advance Peace may be an effective strategy to reduce gun violence.

## Introduction

Community gun violence impacts the health and well-being of neighborhoods and cities [1]. Urban gun violence remains a major driver of racial and ethnic health inequities and is a leading cause of death for Black men under 35 years of age in the United States [2,3]. For example, in 2020, the national homicide rate for Black individuals was 23.41 per 100,000 persons, which was four times greater than the overall homicide rate of 6.03 per 100,000 persons, and seven times greater than the homicide rate for White individuals of 3.24 per 100,000 persons [4]. Among these homicides of Black individuals, 89% were killed with guns [4]. While the rate of gun homicide impacting Black persons has remained relatively stable over the last twenty years, 2020 saw the highest gun homicide rate for Black individuals, demonstrating the increased urgency of this health inequity [5]. A public health approach to ending urban gun violence means centering those most impacted by violence to generate solutions, supporting people and communities to heal from and prevent additional trauma, and eliminating the root causes of racial and economic inequities that can further perpetuate violence [6–8].

A community-driven program, Advance Peace, may be an effective strategy to reduce gun violence in Fresno, California, a city that has seen elevated rates of gun-related homicides and non-fatal firearm injuries, as well as stark racial inequities in gun violence victimization. In 2020, the homicide rate was 14.5 incidents per 100,000 persons, nearly three times greater than the rate for the state of California [9]. There are persistent racial and ethnic inequities in gun violence victimization in Fresno. According to the Fresno Police Department, Black and Latino individuals made up 77% of firearm homicide victims in 2019 and 81% in 2020 [10]. Furthermore, Black men under 35 years old made up 23% of all gun homicide victims [10]. The Advance Peace intervention is organized around the Peacemaker Fellowship, where formerly incarcerated community residents are trained to be street outreach workers, known as neighborhood change agents (NCAs), who engage with individuals who are the most likely to commit gun-related crimes in a city, offer them daily mentorship, social services, and other life-affirming supports, all with the aim of turning them away from gun use. The Advance Peace outreach team spent six months identifying and recruiting these individuals. Because gun violence is often concentrated within a small group of people, targeted outreach to enroll people who hold the most influence over gun use in a city can be effective in curbing gun violence [11,12]. Once recruited, the NCA will work with fellows to develop a Life Management Action Plan, which helps the fellow set short, medium, and long-term goals for personal safety, housing, education, employment, anger management, and conflict resolution. Advance Peace uses a trauma-informed and healing-centered approach to stopping urban gun violence, recognizing and treating the traumas that urban youth have experienced, which may influence them to make fatal decisions, through individual-level mentorship and program support [6]. NCAs also engage in mediating imminent gun conflicts and confront potential violence in the community. Additional details about this program, including its trauma-informed and healing-centered principles, are described previously in the literature [6]. Started in 2010, Advance Peace is one of a relatively new suite of urban, public health-oriented firearm violence prevention strategies that fall under the broader umbrella of community gun violence intervention (CVI), designed to address firearm violence using a trauma-informed approach that builds positive interpersonal and community relationships, promotes access to resources, and increases accountability and capacity to end the cycle of violence [13–16].

The Biden Administration announced the White House Community Violence Intervention Collaborative, where they have described CVI programs as “effective” because they “leverage trusted messengers who work directly with individuals most likely to commit gun violence, intervene in conflicts, and connect people to social, health and wellness, and economic services to reduce the likelihood of violence as an answer to conflict” [17]. CVI, including Advance Peace and Cure Violence, and focused deterrence programs, such as CeaseFire, all build upon decades of research highlighting that urban gun violence is concentrated among a very small number of people and in places that experience racial segregation and disinvestment [16,18]. These interventions are aimed at reducing the prevalence of and preventing future firearm homicides and non-fatal, injury shootings. However, distinguishing between CVI and focused deterrence are essential for understanding the Advance Peace intervention. First, while focused deterrence and CVI both use street outreach workers to engage those with a violent criminal history, CVI, and specifically Advance Peace, focuses exclusively on people who are known to engage in or are actively engaging in gun violence, while focused deterrence targets those who are committing crimes or with a gang or group affiliation. Second, Advance Peace begins with building a trusting relationship with those engaged in shooting without any involvement of law enforcement, whereas focused deterrence works closely with law enforcement to deliver messages around sanctions [6,19]. Third, the services Advance Peace offers are tailored to the individual participant’s needs, as identified by their mentor, not a one-size-fits-all program of employment, mental health counseling, or other social supports, which tends to be the case in focused deterrence programs. Finally, CVI may include but does not primarily emphasize community messaging aimed at de-normalizing gun violence, which is a primary goal of focused deterrence programs [20].

Evaluations of CVI in urban settings have shown mixed success in reducing firearm homicides and injury shootings. For example, the Cure Violence program that was first implemented in Baltimore, Maryland, in 2007, called ‘Safe Streets’, generated inconclusive results as to whether firearm homicides and shootings declined in neighborhoods receiving the intervention [21]. Another Cure Violence program in New York City that was implemented in a select number of high-gun-crime neighborhoods found that the intervention helped reduce gun violence [22]. However, the Rapid Employment and Development Initiative in Chicago, another CVI program, did not result in significant reductions in firearm homicides or shootings [23]. An evaluation of the Advance Peace, Peacemaker Fellowship in Richmond, CA, found that the initiative resulted in a 55% reduction in firearm homicides over a six-year period [24]. However, more evidence is necessary to understand if the Advance Peace approach can be scaled up and replicated in different urban contexts.

In this study, we analyzed the Advance Peace program’s impact on rates of urban firearm homicides and non-fatal injury shootings in Fresno, California. We hypothesized that after the program implementation, there would be a reduction in the rates of gun-related homicides and assaults.

## Methods

### Data

Crime data was obtained from the City of Fresno Police Department from January 2014 to June 2023, which included city-wide geolocated data for gun homicides (California Criminal Code 187a) and firearm assaults (CA Criminal Code 245a2). The homicide file included incident date, location, and victim’s age, sex, and race/ethnicity. The shooting file only included the incident date and location. For this analysis, we aggregated this count data into quarterly counts of total gun-related homicides and assaults, gun-related homicides only, and gun-related assaults only. We chose quarterly as the aggregation level to ensure relatively stable counts while still maintaining variation. To compute rates, yearly population count estimates for the city of Fresno were obtained from the respective American Community Survey 5-year Estimates, using Place, a geography the Census Bureau uses to refer to most cities, some towns, villages, and boroughs, as the census geography for Fresno. Study protocols were approved by the Berkeley Institutional Review Board (Protocol number: 2018-05-11073). The study data is available upon request from the Fresno Police Department.

### Fresno, California

Fresno has long been plagued by high rates of gun homicides and non-fatal firearm injuries. To address this community issue, the Fresno Economic Opportunities Commission began to implement the Advance Peace program starting July 1, 2021, continuing through June 30, 2023 [8]. Advance Peace may be an effective strategy to reduce gun violence in Fresno. While the Advance Peace programming served the entire city of Fresno, it strategically targeted the Southwest neighborhood, a historically disinvested neighborhood with high rates of shootings.

### Advance peace intervention

**Fig 1:**
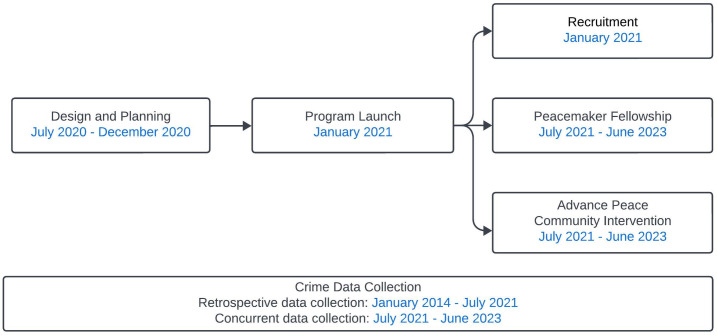
Advance Peace Schematic, Fresno, California, 2020-2023.

Fig 1 demonstrates the timeline of Advance Peace implementation process in Fresno. During the program implementation period, five street outreach workers recruited and enrolled 35 people, called fellows, into the fellowship. Each street outreach worker mentored seven fellows. Each fellow benefitted from daily mentorship provided by their outreach worker. Additionally, they received assistance in navigating various social services, housing, and bureaucratic processes. The program also provided cognitive behavioral therapy, trauma recovery, and culturally responsive counseling. Fellows attended group classes focused on life skills, such as education, job, and financial management skills. Another component of the Advance Peace program involved street outreach workers interrupting conflicts in the community.

### Statistical analysis

Quarterly counts (*n = *38) of total firearm incidents (homicides plus non-fatal assaults), firearm homicides, and firearm-related assaults spanning from the first quarter of 2014 through the second quarter of 2023 were analyzed with separate negative-binomial interrupted time-series (ITS) models, a quasi-experimental design well-suited for policy evaluations when randomization is infeasible [25,26]. Over-dispersion justified the use of the negative-binomial link, and the natural logarithm of the estimated population size was modeled as an offset term to express the results as rates per 100,000 residents. We compared the observed post-intervention trajectory with the counterfactual extension of the pre-intervention trend, utilizing a modern ITS framework [27].

We first parameterized time with restricted cubic splines (RCS) because they offer the same flexibility as higher-order polynomials while avoiding unrealistic oscillations at the tails [28]. The pre-intervention trend was captured with an RCS in calendar time with four degrees of freedom (interior knots positioned at the 5th, 35th, 65th, and 95th percentiles) for the observed baseline trend, following empirical guidance for sample sizes < 100 [28]. Given that Advance Peace launched in the third quarter of 2021 (quarter 31), we developed the following:


$${is\_post}_{t}=\;\left\{ \begin{array}{c} 0,\;\;t<31\\ 1,\;\;t\geq 31 \end{array}\right.\;\;\;\;{and\;\;\;post\_time}_{t}=\max(t-30,\;0).$$

We formed a second RCS basis in the time since the intervention (post_time_*t*_) with three degrees of freedom (knots placed at the 25^th^ and 75^th^ percentiles of the positive values of post_time_*t*_), thus allowing the post‐intervention trajectory to deviate smoothly from the prior trend. Letting ${pop}_{t}$ denote the quarterly population estimate, our full ITS model for the mean count μ_t_ took the form below:


$$\log\left(\mu_{t}\right)=log\left({pop}_{t}\right)+\beta_{0}+\sum_{j=1}^{3}\beta_{j}\;B_{j}\left(t;\;\kappa_{1-4}\right)+\gamma \;{is\_post}_{t}+\sum_{k=1}^{2}\delta_{k}\;C_{k}\left({post\_time}_{t};\;\kappa_{1-2}^{*}\right),$$

where *B*_*j*_ and $C_{k}$ are the RCS basis functions defined by their respective knot sets κ. Given that *B*_*j*_ is evaluated for all 38 quarters and $C_{k}$ multiplies post_time_*t*_ (which is zero before AP began), the two-piece formulation permits curvature both before and after the intervention while avoiding an explicit spline by predictor interaction, yielding the same flexibility as a fully interacted spline, but with fewer parameters to offset our modest sample size. The functions $B_{j}\left(t;\;\kappa_{1-4}\right)$ form a three‐piece restricted cubic spline basis in calendar time, capturing any smooth pre‐intervention trend; the single coefficient γ on ${is\_post}_{t}$ represents an immediate level change at quarter 31. Lastly, we generated 2,000 parametric bootstrap draws of the estimated coefficient vector, recalculated the fitted rates and associated rate ratios for each draw, and defined 95% confidence intervals as the 2.5th and 97.5th percentiles of the resulting empirical distribution. All analyses were conducted in R (version 4.5.0) using the “MASS” package for negative‐binomial regression and the “splines” package for restricted cubic splines.

### Sensitivity analyses

We repeated these analyses using monthly data (January 2014 – June 2023; n = 114 months) to verify that the quarterly findings were not a manifestation of temporal aggregation. The monthly specification retained the same two-piece restricted-cubic-spline structure (four interior knots for calendar time; two interior knots for “time since intervention”), the log‐population offset, and the parametric bootstrap procedure (2,000 draws) used in the primary analysis. Two additional sets of quarterly sensitivity models were fitted to probe potential confounding. Seasonality-adjusted models appended the first harmonic pair of Fourier terms, sin (2πt/4) and cos (2πt/4), to capture recurring annual fluctuations in gun violence. COVID-19–adjusted models added a binary indicator for the pandemic period (2020 Q2 – 2023 Q1) to account for the transient surge in firearm crime observed nationally during that interval. We chose 2023 Q1 as the end point because the California state government declared the end to COVID-19 state of emergency in February 2023. All sensitivity models preserved the core spline specification described above. Counterfactual trajectories and post-intervention rate ratios were generated exactly as in the primary analysis, with 95% confidence intervals obtained from the empirical 2.5th and 97.5th percentiles of the bootstrap distribution.

## Results

**Fig 2:**
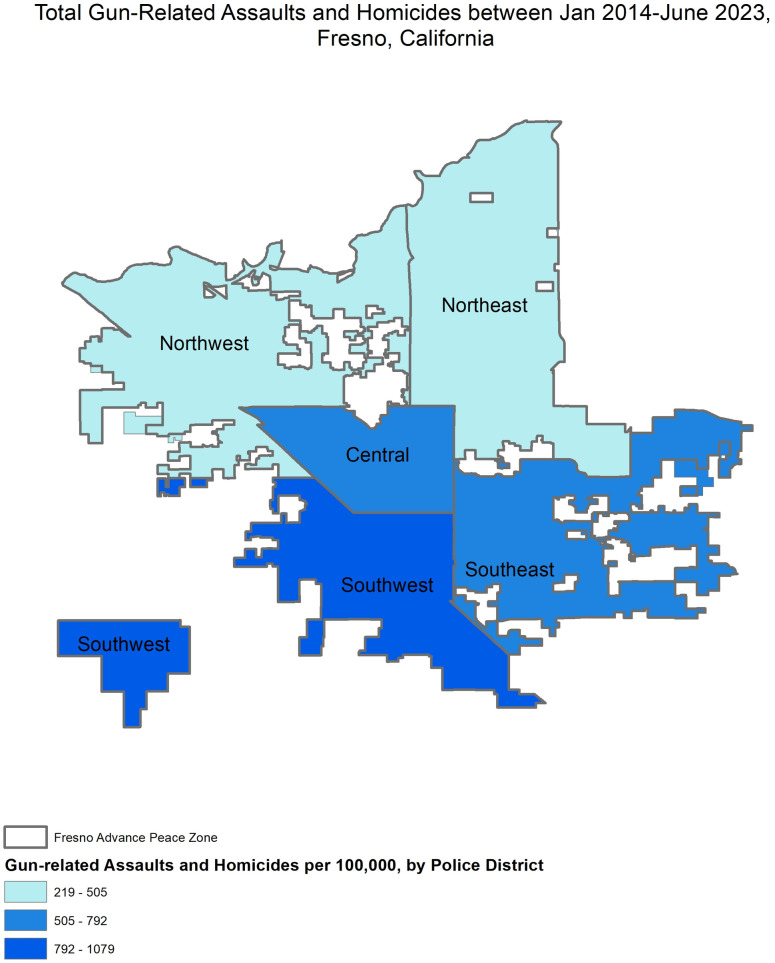
Map of Fresno with Crime Counts, January 2014- June 2023. Figure Label: Source: United States Census Bureau. 2020. TIGER/Line Shapefiles (machine readable data files). U.S. Department of Commerce.

Descriptive analysis showed that during the pre-intervention period, the average quarterly rate of total gun-related crime was the highest during 2020 and 2017, or 17.30 and 13.00 incidents per 100,000 persons, respectively (Table 1). Following the implementation of Advance Peace, the average quarterly rate decreased from 16.29 incidents per 100,000 persons in 2021 to 8.91 incidents per 100,000 persons in 2023. Similarly, for gun-related homicides and gun-related assaults alone, rates were also the highest in 2020, and decreased in the following years between 2021–2023. Geographically, from January 2014 to June 2023, the highest concentration of gun-related homicides and assaults took place in the Southwest district, followed by the Central and Southeast district in Fresno (Fig 2).

**Table 1:** Descriptive statistics of average quarterly gun crime rate, January 2014-June 2023.

	Pre-Intervention							Post-Intervention		
Year	2014	2015	2016	2017	2018	2019	2020	2021	2022	2023
Total	8.79	11.41	12.65	13.00	8.81	9.62	17.30	16.29	12.07	8.91
Homicides	1.88	1.57	1.07	1.93	1.24	1.67	3.28	3.06	2.13	0.93
Assaults	6.92	9.84	11.58	11.08	7.56	7.95	14.02	13.23	9.93	7.98

*Average quarterly rate per 100,000 population.

**Fig 3:**
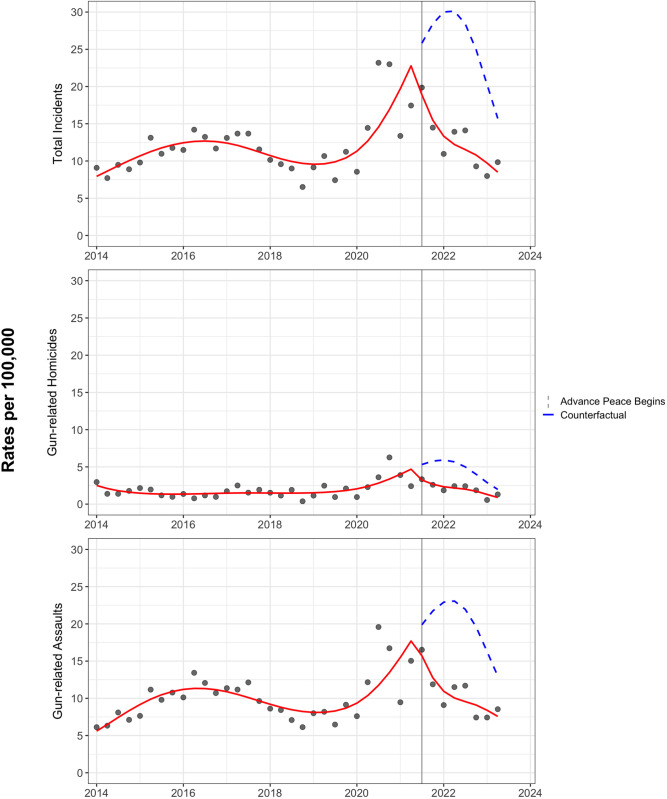
Interrupted Time Series Analysis of Crime Rates, Fresno, California, January 2014- June 2023.

Using the four-knot restricted-cubic-spline specification for calendar time and the two-knot spline for “time since intervention,” we compared observed post‑intervention rates to counterfactual projections at each quarter. Table 2 displays the post-intervention RRs and 95% bootstrap CIs for each quarter from the third quarter of 2021 through the second quarter of 2023. Although not statistically significant, immediately 3 months post-intervention (2021 Q3), the RR for total firearm incidents was 0.74 (95% CI, 0.48–1.11), indicating a 26% reduction from the counterfactual prediction. The downward trajectory continued until the end of Year 2, where the RR reached 0.54 (95% CI, 0.36–0.81), corresponding to a 46% reduction in total firearm incidents relative to counterfactual expectations. Gun‑related homicides followed a similar pattern. Although the earliest two quarterly comparisons were imprecise (Q3 2021 RR = 0.61, 95% CI 0.29–1.31;), by Q2 2022, the RR had fallen to 0.38 (95% CI, 0.16–0.94) and remained < 0.55 for every subsequent quarter. For nonfatal gun assaults, the RR in 2021 Q3 was 0.79 (95% CI, 0.53–1.20), dropping to 0.59 (95% CI, 0.40–0.89) in 2021 Q4. Between Q1 2022 and Q2 2023, all quarterly RRs were <0.60 and statistically significant, where Q2 2023 showed a 42% reduction (RR = 0.58; 95% CI = 0.38–0.89). Fig 3 displays the observed rates together with the spline-based fitted values and counterfactual projections recommended for visualizing ITS estimates [25,27].Visual inspection corroborates the numerical results, as each outcome diverges sharply from its pre-intervention trajectory, beginning in late 2021, and remains below the counterfactual band through June 2023 (Fig 3) .

**Table 2:** Interrupted Time Series Modeling using Quarterly Crime Count in Fresno, CA, January 2014- June 2023.

		Total	Gun Homicides	Gun Assaults
	Time post-intervention	Estimate (95% CI)	Estimate (95% CI)	Estimate (95% CI)
Rate ratios	3 months (1 quarter)	0.74 (0.48-1.11)	0.61 (0.29-1.31)	0.79 (0.53-1.20)
	6 months (2 quarters)	0.55 (0.37-0.80)	0.46 (0.22-0.95)	0.59 (0.40-0.89)
	1 year (4 quarters)	0.41 (0.25-0.66)	0.38 (0.16-0.94)	0.44 (0.27-0.73)
	2 years (8 quarters)	0.54 (0.36-0.81)	0.45 (0.21-0.95)	0.58 (0.38-0.89)

In sensitivity analyses, results remain similar after adjusting for seasonality and using monthly time series of crime count (S1 and S2 Tables). After adjusting for COVID-19 pandemic, results were slightly attenuated, but program effects were statistically significant by the end of Year 1. Specifically, one year after the intervention, there was a decrease in the rate of total gun-related crimes (RR = 0.52, 95% CI: 0.32–0.83), as well as a decrease in gun-related assaults (RR = 0.56, 95% CI: 0.34–0.91) (S3 Table).

## Discussion

This study quantitively assessed trends in gun-related homicides and assaults before and after the implementation of the Advance Peace program in Fresno, California. Leveraging an ITS design, this analysis showed that compared to a hypothetical counterfactual in which Advance Peace was not implemented, this program was associated with a statistically significant reduction in total gun-related crimes, as well as gun-related homicides and assaults. These findings suggest that Advance Peace may be an effective strategy that reduces community gun violence by centering a trauma-informed approach to engage with those who are most impacted by this cycle of violence and provide structured support to improve their lives.

Our findings build on the mixed findings from past evaluations of CVI programs, specifically Cure Violence (formerly Cease Fire). In past studies, some have reported that CVI programs were associated with an initial reduction in homicides. For example, a report evaluating New York’s Cure Violence programs found that the implementation of these programs was associated with a reduction in gun injuries, while an analysis of Newark’s Cease Fire program showed no statistically significant reduction in gun-shot wounds in treatment zones [22,29]. In California specifically, studies conducted in Richmond and Oakland have more consistently reported that the CVI programming was associated with reductions in firearm crimes and shootings [11,24]. Our study yielded results more consistent with studies documenting that CVI programs may lead to a reduction in firearm homicides and injuries. These mixed results suggest the complexity of evaluating the effectiveness of CVI programs and highlight the need for a nuanced understanding of contextual factors that may influence program outcomes. In Fresno, the reduction in firearm homicides and assaults may be attributed to the street outreach workers’ effort to interrupt potentially violent conflicts and prevent retaliatory shootings in Fresno’s communities with higher rates of gun related homicides, and past studies have described how violence interruption may be an important strategy to curb gun violence [21,30].

Furthermore, we hypothesized that the impact of Advance Peace would intensify over time as the program is actively implemented. In other words, there may be a lag between the treatment start date and the point at which the effects of the intervention would be the most pronounced. Results showed that the reduction in gun-related crimes became more pronounced as time passed. For example, after three months post intervention, the estimates showed a decrease in crime rates compared to rates absent the implementation of Advance Peace, but they were not statistically significant. By year 1, the estimates continued to decrease, and the confidence intervals showed statistical significance. By year 2, the magnitude of the reduction had increased, demonstrating that the program’s effects strengthened over time and highlighting the importance of sustained engagement, mentorship, and support for participants.

There are several limitations to this study. First, there may be other drivers that influenced the observed change in firearm crimes, such as neighborhood sociodemographic changes, firearm availability and regulation, and the onset of the COVID pandemic [31]. Sociopolitical events that occurred during this study period, including the 2016 presidential elections and highly visible cases of fatal police killings, may have also impacted communities in Fresno. We relied on data reported by the Fresno police department, which may have reporting error that resulted in bias in the count of crime. However, the Advance Peace team verified count data by sharing the aggregated summary data with the Police Department for verification and confirming aggregated counts based on the Fresno Police Department’s annual Uniform Crime Report to the Federal Bureau of Investigation Crime Data Explorer. Another limitation was that we assessed firearm crime in the entire city of Fresno, which may have heterogeneity in shooting activities, especially given existing evidence that gun violence tends to impact more marginalized neighborhoods. However, the Advance Peace activities were not restricted to a specific neighborhood in the city, and though the activities were more concentrated in the structurally marginalized Southwest region, the impact can also spillover to other areas of the city [11]. Future work can consider the spatial patterning of gun violence and community interventions. Due to the lack of crime data from other cities and difficulty in identifying relevant comparison cities due to state- and federal-level spending on gun violence prevention programming, we were unable to provide a comparison control group, which may help strengthen the results. Post-COVID pandemic, crime rates in other cities in California’s Central Valley saw mixed trend in gun violence between 2019–2023, with the number of shootings increasing in Stockton and Sacramento, and decreasing in Modesto and Bakersfield [32]. Future studies can address this limitation by leveraging more robust crime data and methodologies such as synthetic control or difference-in-difference to build upon our findings. With a longer follow-up period, future studies can also assess the long-term impact of Advance Peace in Fresno.

Despite these limitations, this study provides insightful evidence adding to the body of literature assessing the effectiveness of community-driven gun violence interruption programs. In conclusion, this study provides preliminary evidence that Advance Peace programming may be associated with reductions in gun-related homicides and non-fatal shootings. Results from this study should be considered in conjunction with qualitative evidence documenting the impact of Advance Peace on the lives of those most entrenched in the cycle of gun violence, and their communities. Furthermore, the implementation and impact of Advance Peace should be situated within the larger context of structural racism that affects the distribution of neighborhood resources, economic opportunities, and policy environment shaping the gun violence public health crisis in the United States [33,34].

## Supporting information

S1 Table Interrupted Time Series Modeling using Quarterly Gun Crime Count in Fresno, CA, with Seasonality Adjustment, 2014-2023. (DOCX)

S2 Table Interrupted Time Series Modeling using Monthly Gun Crime Count in Fresno, CA, 2014-2023. (DOCX)

S3 Table Interrupted Time Series Modeling using Quarterly Crime Count in Fresno, CA, with COVID Pandemic Adjustment, 2014-2023. (DOCX)
