# Correction: Evaluating advance peace in Fresno, California: An interrupted times series analysis of a community-based gun violence intervention

**DOI:** 10.1371/journal.pone.0337936

**Published:** 2025-12-02

**Authors:** Xing Gao, Juan R. Cabrera, David Padilla, Mahasin S. Mujahid, Jason Corburn

The word “time” is misspelled, and the name “Advance Peace” is incorrectly capitalized in the article title. The correct title is: Evaluating Advance Peace in Fresno, California: An interrupted time series analysis of a community-based gun violence intervention. The correct citation is: Gao X, Cabrera JR, Padilla D, Mujahid MS, Corburn J (2025) Evaluating Advance Peace in Fresno, California: An interrupted time series analysis of a community-based gun violence intervention. PLoS One 20(8): e0328780. https://doi.org/10.1371/journal.pone.0328780
