# Supplementary material for: Evaluating advance peace in Fresno, California: An interrupted times series analysis of a community-based gun violence intervention
Source: PLoS One. 2025 Aug 27;20(8):e0328780. doi: 10.1371/journal.pone.0328780 (PMC12385352; doi:10.1371/journal.pone.0328780)
Supplement: S1 Table — (DOCX) [file pone.0328780.s001.docx]

Supplemental Table 1: Interrupted Time Series Modeling using Quarterly Gun Crime Count in Fresno, CA, with Seasonality Adjustment, 2014-2023

|  |  | Total | Gun Homicides | Gun Assaults |
| --- | --- | --- | --- | --- |
|  | Time post-intervention | Estimate  (95% CI) | Estimate  (95% CI) | Estimate  (95% CI) |
| Rate ratios | 3 months (1 quarter) | 0.67  (0.44-1.01) | 0.55  (0.26-1.20) | 0.73  (0.49-1.06) |
|  | 6 months (2 quarters) | 0.54  (0.37-0.77) | 0.44  (0.21-0.88) | 0.59  (0.41-0.82) |
|  | 1 year (4 quarters) | 0.41  (0.26–0.64) | 0.37  (0.15-0.87) | 0.45  (0.29-0.68) |
|  | 2 years (8 quarters) | 0.54  (0.37–0.79) | 0.44  (0.21-0.89) | 0.59  (0.41-0.84) |

Models adjusted for seasonality Fourier term
