# Supplementary material for: Evaluating advance peace in Fresno, California: An interrupted times series analysis of a community-based gun violence intervention
Source: PLoS One. 2025 Aug 27;20(8):e0328780. doi: 10.1371/journal.pone.0328780 (PMC12385352; doi:10.1371/journal.pone.0328780)
Supplement: S2 Table — (DOCX) [file pone.0328780.s002.docx]

Supplemental Table 2: Interrupted Time Series Modeling using Monthly Gun Crime Count in Fresno, CA, 2014-2023

|  |  | Total | Gun Homicides | Gun Assaults |
| --- | --- | --- | --- | --- |
|  | Time post-intervention | Estimate  (95% CI) | Estimate  (95% CI) | Estimate  (95% CI) |
| Rate ratios | 3 months (1 quarter) | 0.67  (0.47-0.91) | 0.56  (0.29-1.02) | 0.72  (0.50–0.99) |
|  | 6 months (2 quarters) | 0.50  (0.35-0.71) | 0.43  (0.21-0.82) | 0.54  (0.37-0.77) |
|  | 1 year (4 quarters) | 0.39  (0.25-0.61) | 0.37  (0.16-0.88) | 0.43  (0.27-0.65) |
|  | 2 years (8 quarters) | 0.52  (0.36-0.74) | 0.43  (0.21–0.85) | 0.56  (0.38-0.81) |
