# Supplementary material for: Evaluating advance peace in Fresno, California: An interrupted times series analysis of a community-based gun violence intervention
Source: PLoS One. 2025 Aug 27;20(8):e0328780. doi: 10.1371/journal.pone.0328780 (PMC12385352; doi:10.1371/journal.pone.0328780)
Supplement: S3 Table — (DOCX) [file pone.0328780.s003.docx]

Supplemental Table 3: Interrupted Time Series Modeling using Quarterly Crime Count in Fresno, CA, with COVID Pandemic Adjustment, 2014-2023

|  |  | Total | Gun Homicides | Gun Assaults |
| --- | --- | --- | --- | --- |
|  | Time post-intervention | Estimate  (95% CI) | Estimate  (95% CI) | Estimate  (95% CI) |
| Rate ratios | 3 months (1 quarter) | 0.87  (0.59-1.33) | 0.73  (0.35-1.57) | 0.94  (0.63-1.43) |
|  | 6 months (2 quarters) | 0.72  (0.48-1.08) | 0.61  (0.29-1.29) | 0.77  (0.51-1.19) |
|  | 1 year (4 quarters) | 0.52  (0.32-0.83) | 0.50  (0.19-1.18) | 0.56  (0.34–0.91) |
|  | 2 years (8 quarters) | 0.74  (0.47-1.13) | 0.62  (0.27-1.35) | 0.78  (0.51–1.22) |

Models adjusted for COVID pandemic (March 2020-February 2023)
